# Kidney Transplant: More than Immunological Problems

**DOI:** 10.3390/jcm14062101

**Published:** 2025-03-19

**Authors:** Rosana Gelpi, Angela Casas, Omar Taco, Maya Sanchez-Baya, Mohamed Nassiri, Mónica Bolufer, Javier Paul, Maria Molina, Laura Cañas, Anna Vila, Jordi Ara, Jordi Bover

**Affiliations:** 1 Department of Nephrology, University Hospital Germans Trias i Pujol (HGTiP), 08916 Badalona, Spain; aicasasp.germanstrias@gencat.cat (A.C.); oetaco.germanstrias@gencat.cat (O.T.); mayasanchezb.germanstrias@gencat.cat (M.S.-B.); mnassirin.germanstrias@gencat.cat (M.N.); mboluferc.germanstrias@gencat.cat (M.B.); javierpaulmartinez@gmail.com (J.P.); mmolinago.germanstrias@gencat.cat (M.M.); lacanas.germanstrias@gencat.cat (L.C.); annavilas.germanstrias@gencat.cat (A.V.); jordiaradelrey.germanstrias@gencat.cat (J.A.); jbover.ics@gencat.cat (J.B.); 2 Redes de Investigación Cooperativa Orientadas a Resultados en Salud (RICORS) 2040, 28029 Badalona, Spain; 3 Germans Trias i Pujol Health Sciences Research Institute (IGTP), 08916 Badalona, Spain

**Keywords:** kidney transplantation, chronic kidney disease, cardio-metabolic syndrome, cardiovascular risk, post-transplant diabetes, dyslipidemia, hypertension, post-transplant anemia

## Abstract

Kidney transplantation (KT) represents a pivotal intervention for patients with chronic kidney disease (CKD), significantly improving survival and quality of life. However, KT recipients face an array of non-immunological complications, collectively amplifying cardiovascular (CV) and metabolic risks. This review explores the intersection of cardio-metabolic syndrome and KT, emphasizing the recently introduced cardiovascular–kidney–metabolic (CKM) syndrome. CKM syndrome integrates metabolic risk factors, CKD, and CV disease, with KT recipients uniquely predisposed due to immunosuppressive therapies and pre-existing CKD-related risks. Key issues include post-transplant hypertension, obesity, dyslipidemia, post-transplant diabetes mellitus (PTDM), and anemia. Immunosuppressive agents such as corticosteroids, calcineurin inhibitors, and mTOR inhibitors contribute significantly to these complications, exacerbating metabolic dysfunction, insulin resistance, and lipid abnormalities. For instance, corticosteroids and calcineurin inhibitors heighten the risk of PTDM, while mTOR inhibitors are strongly associated with dyslipidemia. These pharmacologic effects underscore the need for tailored immunosuppressive strategies. The management of these conditions requires a multifaceted approach, including lifestyle interventions, pharmacological therapies like SGLT2 inhibitors and GLP-1 receptor agonists, and close monitoring. Additionally, emerging therapies hold promise in addressing metabolic complications in KT recipients. Proactive risk stratification and early intervention are essential to mitigating CKM syndrome and improving outcomes. This comprehensive review highlights the importance of integrating cardio-metabolic considerations into KT management, offering insights into optimizing long-term recipient health and graft survival.

## 1. Introduction

Kidney transplant (KT) is the solution for the patient with chronic kidney disease (CKD), and in some way, KT is expected to modify the metabolic status. Nevertheless, 19.6% of the recipients develop metabolic syndrome after transplantation with an increased risk of graft loss and CV events, lowering patient survival [1].

The cardiovascular–kidney–metabolic (CKM) syndrome concept, recently incorporated by the American Heart Association (AHA), refers to a systemic dysfunction that reflects the connections between metabolic risk factors, chronic kidney disease (CKD), and the cardiovascular (CV) system The syndrome includes CV events as heart failure, atrial fibrillation, coronary heart disease, stroke, and peripheral arterial disease [2].

Although the detrimental effects of diabetes, cardiovascular risk, and their association with chronic kidney disease (CKD) are well established, chronic kidney management (CKM) presents an innovative approach based on the hypothesis that excessive or dysfunctional adiposity may play a key role in its development [3].

Besides classical CV risk factors, there are additional CV risk factors related to transplantation and immunosuppression (Table 1). Conventional risk factors such as obesity, diabetes, hypertension, and dyslipidemia are common in recipients. Additionally, they also have risk factors associated with pre-existing chronic kidney disease (CKD), inflammatory status, anemia, and proteinuria [4].

Finally, the secondary effects of immunosuppression exacerbate CV outcomes in recipients. Immunosuppressive therapies based on calcineurin inhibitors and corticosteroids, commonly used in most centers, have diabetogenic effects. Steroids also raise cholesterol and LDL levels, as do calcineurin inhibitors and anti-mTOR drugs [4]. Early stratification and treatment of CKM syndrome could help slow the progression of cardiovascular complications.

In the field of transplantation, taking CV risk factors into account is useful not only to perform early treatment but also to adapt the immunosuppressive strategy according to the immunological status and, if possible, according to the stage of the CKM syndrome.

This article reviews the current state of knowledge of the diagnosis, prevention, and treatment of main metabolic and CV risk factors in KRs.

## 2. Hypertension in Kidney Transplantation

Hypertension in KRs has a multifactorial pathogenesis involving traditional risk factors, CKD-related factors, and transplant-specific factors.

Among immunosuppressive treatments, glucocorticoids increase blood pressure (BP) by volume retention and stimulation of the renin–angiotensin–aldosterone system as principal mechanisms.

Calcineurin inhibitors (cyclosporine, tacrolimus) elevate BP through sodium reabsorption and vasoconstriction, though tacrolimus has less pronounced effects compared to cyclosporine. In contrast, purine pathway inhibitors (mycophenolate mofetil, azathioprine) and mTOR inhibitors (everolimus, sirolimus) do not affect BP control [5].

### 2.1. Transplant Renal Artery Stenosis

Transplant renal artery stenosis is a reversible cause of post-transplant hypertension, occurring in 1–23% of cases, often at the renal artery anastomosis site. It typically presents 3 months to 2 years post-transplant with symptoms such as worsening hypertension, hypokalemia, and kidney function decline when treated with angiotensin-converting enzyme inhibitors (ACE inhibitors) or angiotensin receptor blockers (ARBs). Early detection and intervention are crucial for improving outcomes [5].

### 2.2. Management Strategies

BP targets for hypertension management in KRs are based on CKD data, as specific targets for kidney recipients are lacking. Systolic BP < 130 mmHg is a reasonable target.

Lifestyle modifications and drug combinations are generally recommended.

ACEis/ARBs offer conflicting benefits; a recent meta-analysis showed a 38% reduction in graft loss risk but no significant effect on nonfatal CV outcomes or death, with increased hyperkalemia risk.

Dihydropyridine calcium channel blockers (CCBs), however, have shown consistent benefits, including improved graft survival and the mitigation of calcineurin inhibitor vasoconstriction. In the same meta-analysis, CCBs reduced graft loss risk by 42% and increased GFR by 11.11 mL/min compared to ACEis/ARBs, making them a preferred option in the early post-transplant period. Thiazide/Thiazide-like diuretics are also effective, particularly in countering cyclosporine-mediated sodium retention [5,6].

No data are currently available on the long-term effects of antihypertensive drugs on graft loss.

## 3. Obesity in Kidney Transplantation

The prevalence of obesity among kidney candidates and recipients has increased significantly over the past decades. Approximately 50% of patients gain weight after renal transplantation, with the most substantial increase occurring within the first year [7]. The mean weight gain during the first year post-transplantation is 4.5 kg, with 73.4% of patients experiencing weight gain. From 1990 to 2017, the proportion of kidney transplant recipients who were obese (BMI ≥ 30 kg/m^2^) at the time of transplantation more than tripled, from 10.5% to 32.8% [8].

### 3.1. Pre-Transplant Obesity and Transplant Candidacy

Obesity presents significant challenges in the transplant evaluation process as lower rates of referral and waiting listing for obese patients decrease the likelihood of receiving a kidney transplant and lead to potential exclusion from transplant candidacy based on the BMI alone, with some centers using BMI > 35 kg/m^2^ as a contraindication for listing [9,10].

These findings raise important ethical concerns regarding equitable access to transplantation for obese patients. However, recent studies suggest that transplantation shows a clear survival benefit over dialysis for most obese patients, challenging the practice of BMI-based exclusion [11].

### 3.2. Post-Transplant Outcomes and Complications

Obese recipients, particularly those with BMI ≥ 35 kg/m^2^, have an increased risk of surgical site infections and delayed graft function; on the other hand, the impact on graft survival remains controversial since recent evidence suggests that obese kidney transplant recipients may have comparable long-term outcomes to non-obese recipients [8,9].

Weight gain is common after KT, with the most significant increase occurring within the first year. This weight gain, particularly an increase in visceral fat, may contribute to the development of insulin resistance and increase the risk of diabetes [7].

While specific research on CKM syndrome in the context of obesity and KT is limited, several studies provide insights into the interplay between obesity, cardiovascular risk, and metabolic complications post-transplant:

Obesity is a major risk factor for CV disorders, which account for approximately 17% of deaths in KRs [11].

Post-transplant weight gain, especially in visceral fat, is associated with an increased risk of developing insulin resistance and new-onset diabetes after transplantation.

Some studies suggest that obesity negatively affects graft function, with worse renal function observed even in the overweight range [7].

### 3.3. Management Strategies

Weight loss strategies before transplantation may improve outcomes, although the impact on long-term results is debated [12].

Minimally invasive approaches could reduce complications in obese recipients, and close monitoring for weight gain is essential, especially during the first year [13].

Recent research has focused on the potential of new pharmacological interventions in managing obesity and CKM syndrome in kidney transplant recipients.

SGLT2 inhibitors (SGLT2is) show promise in managing diabetes, obesity, and renal function in kidney transplant recipients (KRs), though they may increase the risk of UTIs (Table 2). GLP-1 receptor analogs (GLP-1RAs) help with weight loss and glycemic control, with potential graft protection. The combination of SGLT2is and GLP-1RAs is being explored for CKM syndrome management in KRs. A detailed discussion on diabetes will follow in the next section [14,15,16].

Obesity presents challenges in kidney transplantation. The long-term effects and the complex interactions between obesity, cardiovascular risk, and metabolic complications within the context of CKM syndrome require further study.

BMI-based exclusion from organ transplantation brings up ethical concerns regarding equity, autonomy, and fairness in resource allocation. While aiming to optimize outcomes, this practice could result in discrimination and fail to reflect the overall health of patients. Taking these disparities into account is important to ensure that exclusion criteria do not unintentionally perpetuate global health inequalities.

## 4. Post-Transplant Diabetes Mellitus

Post-transplant diabetes mellitus (PTDM) is a complication of kidney transplantation that affects the patient’s quality of life, survival rate, and kidney graft viability [17,18]. After organ transplantation, screening for hyperglycemia should be performed [19]. The diagnostic criteria for PTDM are similar to those for diabetes in the general population, but the oral glucose tolerance test is the gold standard test to make a diagnosis of PTDM [20]. It is recommended to use glycated hemoglobin (HbA1C) as a diagnostic parameter only after 12 months post-transplant since PTDM may be underdiagnosed due to anemia [20,21,22].

Currently, the term NODAT (new-onset diabetes after transplantation) has been replaced by PTDM, which includes, in addition to those who develop diabetes after transplantation, those who have not been diagnosed before transplantation [23].

The formal diagnosis of PTDM is optimally made once the individual is stable on maintenance immunosuppression (usually at least 45 days after transplantation) and in the absence of acute infection [23].

The lack of a clear definition of PTDM makes prevalence data inconclusive, but it ranges between 4 and 25%. PTDM is most frequently diagnosed between 3 and 6 months post-transplant (early onset) and is related to surgical stress, high doses of corticosteroids, and the initiation of immunosuppressive therapy (especially calcineurin inhibitors). The late onset of PTDM develops after 12 months post-transplant, and its annual incidence is 7%, similar to the prevalence of type 2 diabetes mellitus (T2DM) in the general population [24].

Most studies have reported that transplant patients with PTDM have higher rates of rejection, infection, and re-hospitalization [25].

### 4.1. Pathophysiology of PTDM

The pathophysiological mechanisms involved in the development of PTDM include increased insulin resistance, altered insulin production due to damage to pancreatic beta cells (due to inflammatory factors, oxidative stress, mitochondrial dysfunction, etc.), and uncontrolled release of glucagon [26]. Sometimes, damage to pancreatic beta cells is observed even before transplantation; in fact, 80% of kidney transplant recipients have elevated levels of proinsulin, indicating dysfunction of pancreatic beta cells and increased insulin resistance even before transplantation [27].

Non-modifiable risk factors for PTDM include older age (due to increased adiposity and inflammation that are associated with aging and contribute to insulin resistance), ethnicity (African Americans and Hispanics), male sex, and family history of diabetes (due to genetic predisposition). Modifiable risk factors for PTDM include immunosuppression, rejection episodes, obesity, and hepatitis C virus infection [28,29,30].

Immunosuppressive therapy also contributes to insulin resistance. Corticosteroids have a high dose-dependent diabetogenic potential [31]. Patients receiving immunosuppressive regimens that include corticosteroids have been shown to have a 42% increased risk of developing PTDM [32], and using rapid corticosteroid withdrawal or minimization regimens reduces the incidence of hyperglycemia, dyslipidemia, hypertension, osteoporosis, and cardiovascular complications [33].

Anticalcineurin agents (especially tacrolimus, to a lesser extent cyclosporine) induce apoptosis of pancreatic beta cells, reducing insulin secretion and inducing insulin resistance [34]. Switching from an immunosuppressive regimen based on calcineurin inhibitors to one based on belatacept in kidney transplant patients with diabetes improves glycemic parameters [35].

Regarding mTOR inhibitors, although there are data that sirolimus can cause beta cell injury, no increase in PTDM has been demonstrated with these drugs [36,37].

The evidence does not suggest an increased risk of PTDM with antiproliferative treatment such as mycophenolate mofetil or azathioprine [38].

### 4.2. Management Strategies

The primary treatment of PTDM is insulin therapy, especially in the immediate postoperative period of renal transplant surgery [39]. When insulin resistance is due to corticosteroid treatment in KRs, a combination of rapid-acting insulin with slow-acting insulin is recommended [40].

The use of metformin may be controversial due to the increased risk of acute kidney injury in KRs and the increased risk of lactic acidosis in the immediate postoperative period of renal transplantation [41]. Metformin is limited based on estimated glomerular filtration rate (eGFR) and can only be used if >30 mL/min/1.73 m^2^).

Thiazolidinediones are not recommended in renal transplant recipients due to the increased risk of edema and heart failure [42].

Due to the high risk of hypoglycemia, sulfonylureas should be used with extreme caution, especially if treated with cyclosporine or with drugs that alter sulfonylurea metabolism, such as azoles [43].

Although dipeptidyl peptidase-4 inhibitors are well tolerated and safe, improving glucose metabolism in the transplant population, the beneficial effects of reducing PTDM have not been confirmed [44,45].

SGLT2i shows improvements in cardio renal outcomes in the non-renal transplant population [46,47,48,49]. In PTDM, they reduce weight and HbA1c levels, but it is a slight reduction compared to other pharmacological groups since the reduction is greater the more the eGFR is preserved [50].

GLP-1 RAs reduce the incidence of cardiovascular events and progression of kidney disease in type 2 DM and CKD, but these benefits have not been demonstrated in PTDM [51]. In transplant patients, GLP-1 RAs are safe, reduce the need for exogenous insulin, and do not increase the risk of graft failure [52].

Other drugs could improve and change the management of PTDM: finerenone, tirzepatide, glucokinase activators, dorzagliatin, imeglimin, amycretin, and pramlintide; however, more studies are required to validate them in the transplant population.

## 5. Dyslipidemia in Kidney Transplant

Dyslipidemia (DLP) in kidney recipients is characterized by altered lipid profiles (elevated low-density lipoprotein cholesterol (LDL-C), reduced high-density lipoprotein cholesterol (HDL-C), and elevated triglycerides), and it is associated with an increased risk of cardiovascular disease (CVD), a leading cause of morbidity and mortality in this population [53] (Table 3). DLP can lead to thrombotic disturbances by increasing platelet reactivity. It has been observed that an increase in visceral or subcutaneous adipose tissue is associated with a decrease in graft kidney function. Some studies suggest that elevated total cholesterol (TC) and triglycerides (TGs) can cause podocyte injury and proteinuria in the renal graft. Hypertriglyceridemia is a known cause of pancreatitis, a complication that can be very severe in kidney transplant recipients [53].

The pathogenesis of DLP in KRs is multifactorial, involving both traditional CV risk factors and the effects of immunosuppressive therapy, which plays a fundamental role in the development of DLP.

One of the most significant contributors to DLP are calcineurin inhibitors such as cyclosporine and tacrolimus, which are commonly prescribed as part of the immunosuppressive regimen to prevent graft rejection [54].

Calcineurin inhibitors have been shown to induce hyperlipidemia by increasing the synthesis of LDL-C and reducing the clearance of triglycerides. Studies suggest that tacrolimus, compared to cyclosporine, is associated with less pronounced lipid abnormalities, though it can still contribute to elevated LDL-C levels [54].

Cyclosporin induces dose-dependent alterations in lipid metabolism. It negatively regulates the expression of the LDL receptor, increasing PCSK9 and decreasing the intestinal absorption of cholesterol by inhibiting the enzyme cholesterol 7 alpha-hydroxylase. Tacrolimus induces less DLP than cyclosporine. The conversion from CsA to TAC has been associated with a reduction in lipid concentrations in numerous studies [54].

Glucocorticoids increase hepatic synthesis of VLDL and induce a downregulation of LDL-C, likely modulating the interaction of glucocorticoid receptors at the genetic level. Therefore, they promote an elevation of total cholesterol, LDL-C, triglycerides (TGs), and VLDL. The effect is dose-dependent [55,56].

DLP is one of the most well-known side effects of mTOR inhibitors. This group of IS agents has the highest potential for causing dyslipidemia. The mechanisms are not fully understood, although it is known that they increase the hepatic synthesis of apoB100 and the secretion of VLDL-C and decrease the hepatic catabolism of LDL-C mediated by PCSK9 [57].

They cause an elevation in total cholesterol, LDL-C, non-HDL cholesterol, and particularly in triglycerides and VLDL. This effect is dose-dependent and improves when the dose is reduced or levels are lowered. There are no studies that have directly compared the dyslipidemic effects of sirolimus and everolimus.

On the other hand, nycophenolate mofetil, mycophenolic acid, azathioprine, and induction agents, such as polyclonal anti-lymphocyte antibodies and monoclonal antibodies (anti-CD25, Rituximab), do not alter lipid levels.

Belatacept does not modify lipid levels (Table 4).

### Management Strategies

Effective lipid management is critical for reducing CV and graft failure risks. The management of DLP in TR is comparable to the recommendations for patients at high or very high CV risk, although more attention is needed regarding the causes of the lipid disturbances and possible side effects due to drug–drug interactions [58].

The figure (Table 5) describes the LDL-C goals for kidney recipients.

Dietary interventions, physical activity, and weight management are essential components of dyslipidemia management. A low-fat, high-fiber diet rich in omega-3 fatty acids can help improve lipid profiles in KRs [57].

Statins are the cornerstone of dyslipidemia treatment as they have been shown to lower LDL-C levels and reduce cardiovascular events. Statin therapy not only improves lipid profiles but also reduces the risk of cardiovascular mortality and graft failure [55].

However, the use of statins requires careful monitoring due to potential drug interactions with immunosuppressive agents, especially cyclosporine. Also, it is crucial to monitor for potential side effects, such as liver toxicity or rhabdomyolysis, since statins are primarily metabolized in the liver through the cytochrome P450 system, particularly by the CYP3A4 enzyme. Different statins, like fluvastatin, pravastatin, pitavastatin, and rosuvastatin, utilize various metabolic pathways, which helps minimize drug–drug interactions. While most statins are lipophilic, pravastatin and rosuvastatin are hydrophilic, contributing to their improved safety profiles [56].

Ezetimibe, which inhibits intestinal cholesterol absorption, can be used in combination with statins for patients who do not achieve adequate LDL-C reduction with statins alone. Additionally, recent studies have explored the use of PCSK9 inhibitors in KTRs with familial hypercholesterolemia or statin-intolerant patients [57].

For patients with elevated triglycerides and low HDL-C, fibrates can be considered, although their use in KRs is often limited by the potential for renal toxicity and drug interactions with immunosuppressive medications [58].

## 6. Post-Transplantation Anemia

Post-transplantation anemia (PTA) is not very well defined in terms of degree of anemia or time after transplantation. It is defined by some authors as early and late PTA up or after 6 months from transplantation, respectively [59,60].

PTA is estimated to be in the range of 20% to 40% of patients [61].

There are multiple causes of PTA, but the most common reason for early PTA is iron deficiency, which may be caused by the depletion of iron stores before transplantation and perioperative blood loss [62]. Slowly increasing levels of erythropoietin, especially when delay graft function occurs, may contribute to the anemia. The occurrence of late PTA has been associated with impaired graft function and the development of renal insufficiency [63].

Immunosuppressive agents contribute to post-transplant anemia (PTA), both in the early and late stages. Induction therapies, such as Thymoglobulin, and maintenance medications like mycophenolate mofetil, can lead to bone marrow suppression. Additionally, viral infections, including cytomegalovirus, Epstein–Barr virus, BK virus, and parvovirus, frequently observed in kidney recipients, can directly cause hematological abnormalities.

Several studies have shown that PTA may be associated with increased mortality, decreased graft survival, and de novo congestive heart failure [64].

### Management Strategies

The treatment of PTA should begin immediately after kidney transplantation [65].

The recommended hemoglobin target for kidney transplant recipients is generally between 10 and 12 g/dL in order to avoid both anemia and the risks associated with over-correction. The excessive correction of anemia, with hemoglobin levels exceeding 13 g/dL, has been associated with an increased risk of adverse events, including hypertension, stroke, and thromboembolic events.

This target range for hemoglobin should be adjusted individually based on the patient’s clinical characteristics, including cardiovascular condition and the presence of other comorbidities. Anemia management in these patients is typically achieved with a combination of erythropoiesis-stimulating agents, iron supplementation, and treatment of the underlying cause [65,66,67].

## 7. Summary

An elevated CKM stage is associated with a higher risk of cardiovascular events, cardiovascular death, and allograft [68].

Classifying patients based on CKM helps in identifying patients at higher risk for cardiovascular events, mortality, and graft loss, which may influence clinical decisions for monitoring and management.

The knowledge of the contribution of the immunosuppressive strategy to CKM syndrome can help to choose the best pharmacological combination according to the immunological status and cardio-metabolic risk (Table 6).

## Figures and Tables

**Table 1:** Most important CV and metabolic risk factors.

Traditional CV Risk Factors	CV Risk Factors Related to CKD	CV Risk Factors Related to Transplantation
Genetic predisposition Age Lifestyle Tobacco Obesity Hypertension Diabetes Dislipemia	Uremia CKD stage Chronic inflammation Hyperhomocysteinemia Time in dialysis Proteinuria	Immunosupression drugs Post-transplant diabetes Suboptimal graft function Post-transplant Anemia

**Table 2:** Potential benefits of SGLT2i and GLP-1.

	SGLT2i	GLP-1RA
Glycemic control	+	+
Weight loss	+	+
Renal function: stable or improved eGFR	+	+
BP control	+	-

**Table 3:** DLP in transplant recipients is defined as the presence of one or more of the following criteria.

Total cholesterol	>200 mg/dL
LDL cholesterol	>100 mg/dL
HDL cholesterol	<40 mg/dL (men); <45 mg/dL (women)
Triglycerides	>150 mg/dL
Non-HDL cholesterol	>130 mg/dL

**Table 4:** Potential dyslipidemic effects of major immunosuppressants.

Sirolimus/Everolimus	++++
Steroids	++++
Cyclosporine	+++
Tacrolimus	++
Mycophenolate Mofetil/Mycophenolate Sodium	-
Basiliximab	-
Polyclonal anti-lymphocyte antibodies	-
Belatacept	-

**Table 5:** LDL-C goals.

GFR between 30 and 59 mL/min/1.73 m^2^	<50% LDL-C reduction from baseline	LDL-C goal < 1.8 mmol/L (<70 mg/dL)
GFR < 30 mL/min/1.73 m^2^	≥50% LDL-C reduction from baseline	LDL-C goal < 1.4 mmol/L (<55 mg/dL)

**Table 6:** Main immunosuppressive maintenance drugs and contribution to CV risk.

	BP	PTDM	DLP	PTA
Steroids	++	+++	++++	-
Cyclosporine	+++	++	+++	-
Tacrolimus	++	+++	++	-
Sirolimus/Everolimus	-	-	++++	+
Mycophenolate Mofetil/Mycophenolate Sodium	-	-	-	++
